# Supplementary material for: Modeling glioblastoma heterogeneity as a dynamic network of cell states
Source: Mol Syst Biol. 2021 Sep 16;17(9):e10105. doi: 10.15252/msb.202010105 (PMC8444284; doi:10.15252/msb.202010105)
Supplement: Supplementary file 5 — Source Data for Figure 3 [file MSB-17-e10105-s001.zip › Figure3A_sourcedata/GSEA_3065/hallmarks_state3.GseaPreranked.1623416274744/HALLMARK_EPITHELIAL_MESENCHYMAL_TRANSITION.html]

Details for gene set HALLMARK\_EPITHELIAL\_MESENCHYMAL\_TRANSITION[GSEA]

|  || Dataset | state3 |
| Phenotype | NoPhenotypeAvailable |
| Upregulated in class | na\_pos |
| GeneSet | HALLMARK\_EPITHELIAL\_MESENCHYMAL\_TRANSITION |
| Enrichment Score (ES) | 0.55269706 |
| Normalized Enrichment Score (NES) | 2.2059383 |
| Nominal p-value | 0.0 |
| FDR q-value | 0.0 |
| FWER p-Value | 0.0 |
Table: GSEA Results Summary

  

Fig 1: Enrichment plot: HALLMARK\_EPITHELIAL\_MESENCHYMAL\_TRANSITION      
 Profile of the Running ES Score & Positions of GeneSet Members on the Rank Ordered List

  

| PROBE | GENE SYMBOL | GENE\_TITLE | RANK IN GENE LIST | RANK METRIC SCORE | RUNNING ES | CORE ENRICHMENT || 1 | POSTN |  |  | 24 | 0.487 | 0.0224 | Yes |
| 2 | TIMP1 |  |  | 35 | 0.470 | 0.0454 | Yes |
| 3 | CALU |  |  | 48 | 0.437 | 0.0666 | Yes |
| 4 | TFPI2 |  |  | 65 | 0.388 | 0.0847 | Yes |
| 5 | DST |  |  | 80 | 0.365 | 0.1020 | Yes |
| 6 | GPC1 |  |  | 90 | 0.355 | 0.1192 | Yes |
| 7 | EMP3 |  |  | 91 | 0.354 | 0.1375 | Yes |
| 8 | LAMC1 |  |  | 96 | 0.349 | 0.1550 | Yes |
| 9 | CALD1 |  |  | 124 | 0.322 | 0.1685 | Yes |
| 10 | SGCB |  |  | 133 | 0.317 | 0.1839 | Yes |
| 11 | PPIB |  |  | 142 | 0.313 | 0.1992 | Yes |
| 12 | ITGB1 |  |  | 150 | 0.310 | 0.2144 | Yes |
| 13 | LRP1 |  |  | 152 | 0.309 | 0.2302 | Yes |
| 14 | IGFBP2 |  |  | 164 | 0.303 | 0.2446 | Yes |
| 15 | COL4A2 |  |  | 232 | 0.271 | 0.2508 | Yes |
| 16 | COL4A1 |  |  | 241 | 0.267 | 0.2636 | Yes |
| 17 | TNC |  |  | 249 | 0.264 | 0.2765 | Yes |
| 18 | COL5A2 |  |  | 266 | 0.255 | 0.2877 | Yes |
| 19 | CDH2 |  |  | 283 | 0.249 | 0.2987 | Yes |
| 20 | VCAN |  |  | 293 | 0.245 | 0.3104 | Yes |
| 21 | COL5A1 |  |  | 302 | 0.242 | 0.3219 | Yes |
| 22 | SERPINE2 |  |  | 308 | 0.241 | 0.3338 | Yes |
| 23 | MATN2 |  |  | 318 | 0.239 | 0.3450 | Yes |
| 24 | ITGAV |  |  | 322 | 0.238 | 0.3570 | Yes |
| 25 | THY1 |  |  | 326 | 0.236 | 0.3688 | Yes |
| 26 | SPARC |  |  | 361 | 0.228 | 0.3766 | Yes |
| 27 | FBLN1 |  |  | 365 | 0.227 | 0.3880 | Yes |
| 28 | TNFRSF12A |  |  | 380 | 0.223 | 0.3979 | Yes |
| 29 | PLOD3 |  |  | 443 | 0.210 | 0.4015 | Yes |
| 30 | ADAM12 |  |  | 455 | 0.208 | 0.4110 | Yes |
| 31 | NT5E |  |  | 511 | 0.197 | 0.4148 | Yes |
| 32 | PCOLCE |  |  | 533 | 0.194 | 0.4224 | Yes |
| 33 | PMP22 |  |  | 556 | 0.192 | 0.4297 | Yes |
| 34 | SERPINH1 |  |  | 588 | 0.188 | 0.4358 | Yes |
| 35 | PTX3 |  |  | 605 | 0.185 | 0.4435 | Yes |
| 36 | COLGALT1 |  |  | 630 | 0.182 | 0.4502 | Yes |
| 37 | FLNA |  |  | 633 | 0.181 | 0.4593 | Yes |
| 38 | P3H1 |  |  | 677 | 0.175 | 0.4633 | Yes |
| 39 | PLOD2 |  |  | 683 | 0.173 | 0.4717 | Yes |
| 40 | COL11A1 |  |  | 684 | 0.173 | 0.4806 | Yes |
| 41 | GPX7 |  |  | 705 | 0.169 | 0.4870 | Yes |
| 42 | CD59 |  |  | 713 | 0.168 | 0.4949 | Yes |
| 43 | FBN1 |  |  | 768 | 0.161 | 0.4969 | Yes |
| 44 | SCG2 |  |  | 778 | 0.160 | 0.5042 | Yes |
| 45 | FSTL1 |  |  | 790 | 0.159 | 0.5111 | Yes |
| 46 | LOXL2 |  |  | 817 | 0.156 | 0.5161 | Yes |
| 47 | COPA |  |  | 853 | 0.152 | 0.5199 | Yes |
| 48 | PTHLH |  |  | 854 | 0.152 | 0.5277 | Yes |
| 49 | LAMA1 |  |  | 930 | 0.145 | 0.5265 | Yes |
| 50 | MCM7 |  |  | 949 | 0.143 | 0.5318 | Yes |
| 51 | TIMP3 |  |  | 950 | 0.142 | 0.5392 | Yes |
| 52 | HTRA1 |  |  | 955 | 0.142 | 0.5460 | Yes |
| 53 | FN1 |  |  | 973 | 0.140 | 0.5513 | Yes |
| 54 | ITGB5 |  |  | 1139 | 0.127 | 0.5387 | Yes |
| 55 | GJA1 |  |  | 1160 | 0.125 | 0.5429 | Yes |
| 56 | PLOD1 |  |  | 1185 | 0.122 | 0.5464 | Yes |
| 57 | COL16A1 |  |  | 1199 | 0.122 | 0.5512 | Yes |
| 58 | CDH11 |  |  | 1289 | 0.116 | 0.5468 | Yes |
| 59 | SFRP1 |  |  | 1291 | 0.116 | 0.5527 | Yes |
| 60 | SPP1 |  |  | 1358 | 0.111 | 0.5508 | No |
| 61 | WNT5A |  |  | 1428 | 0.107 | 0.5483 | No |
| 62 | THBS1 |  |  | 1561 | 0.100 | 0.5382 | No |
| 63 | SDC1 |  |  | 1568 | 0.100 | 0.5426 | No |
| 64 | BMP1 |  |  | 1722 | 0.090 | 0.5296 | No |
| 65 | VEGFA |  |  | 1723 | 0.090 | 0.5342 | No |
| 66 | CADM1 |  |  | 1735 | 0.090 | 0.5376 | No |
| 67 | CD44 |  |  | 1748 | 0.089 | 0.5408 | No |
| 68 | MMP2 |  |  | 1898 | 0.081 | 0.5277 | No |
| 69 | NOTCH2 |  |  | 1990 | 0.076 | 0.5211 | No |
| 70 | IGFBP3 |  |  | 2112 | 0.071 | 0.5108 | No |
| 71 | SDC4 |  |  | 2189 | 0.069 | 0.5055 | No |
| 72 | QSOX1 |  |  | 2301 | 0.064 | 0.4960 | No |
| 73 | COL6A2 |  |  | 2565 | 0.055 | 0.4684 | No |
| 74 | ITGA5 |  |  | 2747 | 0.049 | 0.4499 | No |
| 75 | TGFBI |  |  | 2753 | 0.049 | 0.4518 | No |
| 76 | COL5A3 |  |  | 2891 | 0.044 | 0.4383 | No |
| 77 | SERPINE1 |  |  | 3090 | 0.039 | 0.4173 | No |
| 78 | CAP2 |  |  | 3313 | 0.033 | 0.3933 | No |
| 79 | APLP1 |  |  | 3403 | 0.030 | 0.3846 | No |
| 80 | WIPF1 |  |  | 3411 | 0.030 | 0.3853 | No |
| 81 | NNMT |  |  | 3707 | 0.022 | 0.3523 | No |
| 82 | FUCA1 |  |  | 3766 | 0.021 | 0.3467 | No |
| 83 | FERMT2 |  |  | 3823 | 0.020 | 0.3412 | No |
| 84 | LOXL1 |  |  | 4025 | 0.015 | 0.3187 | No |
| 85 | PLAUR |  |  | 4075 | 0.013 | 0.3137 | No |
| 86 | TGFB1 |  |  | 4110 | 0.012 | 0.3104 | No |
| 87 | GAS1 |  |  | 4210 | 0.009 | 0.2994 | No |
| 88 | ENO2 |  |  | 4676 | -0.003 | 0.2457 | No |
| 89 | GLIPR1 |  |  | 5113 | -0.014 | 0.1959 | No |
| 90 | CXCL12 |  |  | 5164 | -0.015 | 0.1909 | No |
| 91 | SNTB1 |  |  | 5409 | -0.021 | 0.1637 | No |
| 92 | TPM1 |  |  | 5672 | -0.029 | 0.1349 | No |
| 93 | NTM |  |  | 5738 | -0.031 | 0.1289 | No |
| 94 | SLC6A8 |  |  | 5778 | -0.032 | 0.1261 | No |
| 95 | TPM2 |  |  | 5823 | -0.034 | 0.1227 | No |
| 96 | MSX1 |  |  | 5833 | -0.034 | 0.1234 | No |
| 97 | BDNF |  |  | 5896 | -0.036 | 0.1181 | No |
| 98 | DKK1 |  |  | 6057 | -0.041 | 0.1017 | No |
| 99 | MYLK |  |  | 6330 | -0.050 | 0.0728 | No |
| 100 | MEST |  |  | 6477 | -0.056 | 0.0587 | No |
| 101 | GADD45B |  |  | 6619 | -0.062 | 0.0456 | No |
| 102 | FGF2 |  |  | 6685 | -0.065 | 0.0414 | No |
| 103 | RHOB |  |  | 6706 | -0.066 | 0.0425 | No |
| 104 | TAGLN |  |  | 6722 | -0.067 | 0.0442 | No |
| 105 | EFEMP2 |  |  | 7457 | -0.113 | -0.0350 | No |
| 106 | PRRX1 |  |  | 7670 | -0.136 | -0.0525 | No |
| 107 | JUN |  |  | 7746 | -0.146 | -0.0537 | No |
| 108 | CAPG |  |  | 7767 | -0.148 | -0.0484 | No |
| 109 | ID2 |  |  | 7934 | -0.174 | -0.0586 | No |
| 110 | PDLIM4 |  |  | 8272 | -0.246 | -0.0850 | No |
| 111 | GADD45A |  |  | 8306 | -0.260 | -0.0754 | No |
| 112 | DPYSL3 |  |  | 8391 | -0.291 | -0.0701 | No |
| 113 | PFN2 |  |  | 8503 | -0.342 | -0.0654 | No |
| 114 | PMEPA1 |  |  | 8520 | -0.346 | -0.0494 | No |
| 115 | SAT1 |  |  | 8533 | -0.354 | -0.0325 | No |
| 116 | TPM4 |  |  | 8594 | -0.396 | -0.0190 | No |
| 117 | LGALS1 |  |  | 8744 | -0.718 | 0.0008 | No |
Table: GSEA details [plain text format]

  

Fig 2: HALLMARK\_EPITHELIAL\_MESENCHYMAL\_TRANSITION: Random ES distribution      
 Gene set null distribution of ES for **HALLMARK\_EPITHELIAL\_MESENCHYMAL\_TRANSITION**
